# The Effects of ML385 on Head and Neck Squamous Cell Carcinoma: Implications for NRF2 Inhibition as a Therapeutic Strategy

**DOI:** 10.3390/ijms25137011

**Published:** 2024-06-27

**Authors:** Eun-Jeong Jeong, Jong Joong Choi, Sun Young Lee, Yeon Soo Kim

**Affiliations:** 1 Department of Otorhinolaryngology, Myunggok Medical Research Institute, Konyang University Hospital, Konyang University College of Medicine, Daejeon 35365, Republic of Koreachoijj824@naver.com (J.J.C.); lsy9906074@naver.com (S.Y.L.); 2 Department of Otorhinolaryngology, Korea University Anam Hospital, Korea University College of Medicine, Seoul 02841, Republic of Korea

**Keywords:** head and neck squamous cell carcinoma (HNSCC), ML385, nuclear factor erythroid 2-related factor 2 (NRF2), cell cycle arrest

## Abstract

Head and neck squamous cell carcinoma (HNSCC) affects squamous cells in the head and neck region and is currently ranked as the sixth most common cancer worldwide. NF-E2-related factor 2 (NRF2) plays a crucial role in cellular protection and defence mechanisms and NRF2 over-expression has been linked to various cancers; however, its role in the response of HNSCC cells remains elusive. We investigated the effects of ML385, a selective NRF2 inhibitor, on HNSCC to understand the underlying molecular mechanisms, and to assess the potential of ML385 as a therapeutic agent. We treated HNSCC cell lines with ML385 and observed a significant reduction in the expression of NRF2 and its downstream target, heme oxygenase-1 (HO-1), using Western blotting. We evaluated its effects on various cellular processes, including cell proliferation, cloning, migration, and wound healing, in HNSCC cell lines. ML385 treatment substantially reduced NRF2 expression, promoting a decrease in the investigated cellular activities. Additionally, we examined changes in the expression of cell-cycle-related proteins and found that ML385 induced cell cycle arrest at the G1/S phase in HNSCC cell lines. Our findings suggest that ML385 can regulate cell cycle progression, inhibit HNSCC growth, and have potential as a therapeutic agent for HNSCC.

## 1. Introduction

Head and neck cancer is a variety of cancer that occurs mainly in the squamous cells lining the mouth, neck, and nose and is called head and neck squamous cell carcinoma (HNSCC). HNSCC is the sixth most common cancer worldwide, accounting for approximately 4% of all cancers and over 500,000 new cases reported annually [1,2,3]. Tobacco smoking, alcohol consumption, and human papillomavirus infection have been identified as potential risk factors for HNSCC [4]. Surgical procedures and non-surgical options, such as radiological therapy, oral robotic surgery, laser surgery, and chemotherapy, are the choices for HNSCC management [5]. Despite intensive research, the prognosis of patients with HNSCC remains poor, resulting in an overall survival rate that has remained relatively unchanged at 50% over the past 30 years [2].

Nuclear factor erythroid 2-related factor 2 (NRF2) is a transcription factor that regulates the expression of several antioxidant proteins. Kelch-like ECH-associated protein-1 (KEAP1) mediates the degradation of NRF2 as an oxidative stress sensor and is a well-known substrate for KEAP1 [6]. In the absence of KEAP1, the expression of NRF2 target genes is structurally stabilised, and their expression is maintained at high levels [6]. Overexpression of NRF2 induces the transcription of various genes that activate antioxidant reactions and combat the harmful effects of oxidative stress. HO-1 (heme oxygenase-1), one of the genes regulated by NRF2, is expressed through the binding of NRF2 to the antioxidant response element (ARE) and plays a crucial role in cellular defence against oxidative stress [7,8]. The KEAP1/NRF2 signalling pathway plays an important role in the cellular responses to oxidative stress, electrical substances, and heterogeneous antibiotics. KEAP1 mutations induce constitutive activation of NRF2, which protects cancer cells from chemotherapeutic drugs. Activated NRF2 by KEAP1 is associated with an increased progression of cancer and reduced survival [9,10,11]. At the intersection of multiple oncogenic and cytoprotective pathways, NRF2 plays a direct or indirect role in each of the cancer hallmarks described thus far, including carcinogenesis, sustained proliferation, evasion of apoptosis, metabolic reprogramming, altered redox balance, formation of metastases, and resistance to therapy [12]. A growing body of evidence indicates that several drug efflux transporters controlled by NRF2 are crucial determinants of therapeutic resistance in many tumours. A quantitative high-permeation screen of the molecular library small-molecule reservoir was performed to identify novel and specific small-molecule inhibitors of NRF2 to reduce treatment resistance in lung cancer. A thiazole–indoline compound series was identified during the screening [13].

The optimisation of medicinal chemistry has led to the development of ML385, a novel inhibitor of NRF2 signalling in non-small cell lung cancer carcinoma (NSCLC) cells. When used in combination with carboplatin, ML385 demonstrates specificity, selectivity, and in vivo efficacy in the NSCLC model [13]. Although little is known about the function of antioxidant enzymes in cancer cells, recent reports have suggested that enzymes such as glutathione S-transferase, glutathione peroxidase, NADH quinine oxidoreductase-1, peroxiredoxins, thioredoxin, and PPIA (cyclophilin A), protect cancer cells against oxidative stress-induced apoptosis, hypoxia, and chemotherapy [14]. However, the specific mechanisms underlying the relationship between oxidative stress, antioxidant enzymes, CD147, and HNSCC remain unclear, and the roles of these proteins in OSCC have not been fully elucidated. Cisplatin is an effective antitumor agent that exhibits broad activity against various human solid tumours, including ovarian, bladder, cervical, head and neck, oesophageal, NSCLC, and gastric cancer [15,16,17,18]. 

However, the effect of ML385 on head and neck cancers has not yet been reported. Therefore, in this study, we hypothesised that ML385 could regulate cell cycle progression, inhibit HNSCC growth, and hold promising potential as a therapeutic agent for the treatment of HNSCC. Our goal was to investigate the effects of ML385 on HNSCC and elucidate the underlying molecular mechanisms.

## 2. Results

### 2.1. Expression of NRF2 in HNSCC

**Figure 1:**
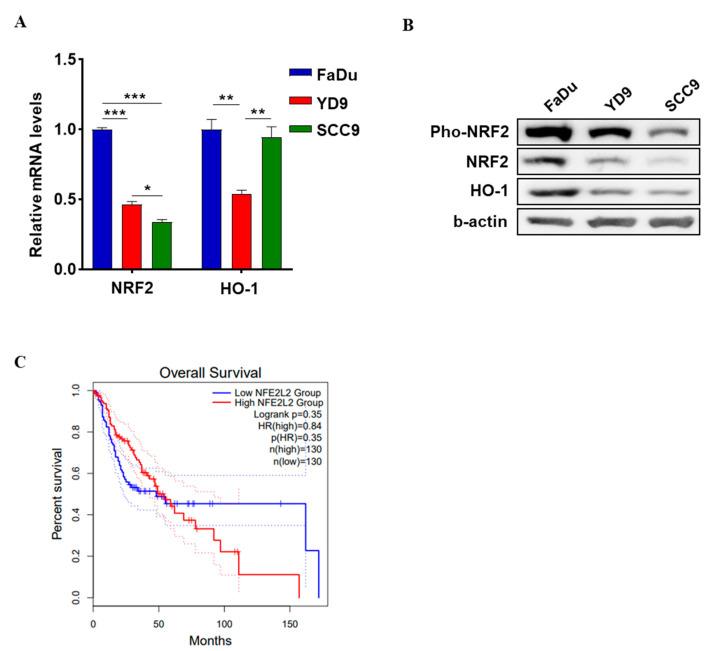
NF-E2-related factor 2 (NRF2) overexpression in head and neck squamous cell carcinoma (HNSCC) cell lines. (**A**) NRF2 mRNA and (**B**) protein expression levels in FaDu, YD9, and SCC9 cell lines. (**C**) Overall survival analysis for head and neck cancer samples from GEPIA depending on NRF2 (NEF2L2) expression. The figure shows that the red solid line represents a high-risk group, while the blue line represents a low-risk group. The solid lines represent the survival curves, while the dashed lines represent the 95% confidence intervals. * *p* < 0.05, ** *p* < 0.01, and *** *p* < 0.001.

NRF2 expression in HNSCC was confirmed using qRT-PCR and Western blotting. NRF2 was found to be phosphorylated in HNSCC cells. Upregulated NRF2 mRNA and protein expression was observed in FaDu and YD9 cells compared to SCC9 cells, and the expression of HO-1, which expression is regulated by NRF2 expression [7,8], was increased by NRF2 (Figure 1A,B). This study validated the overexpression of NRF2 in HNSCC and its association with patient survival using the Gene Expression Profiling Interactive Analysis (GEPIA) database (Figure 1C). Patients with high NRF2 expression had significantly shorter overall survival compared to those with low NRF2 expression. These results suggest that NRF2 is a potential therapeutic target for HNSCC.

### 2.2. ML385 Inhibits the Viability of HNSCC Cells

**Figure 2:**
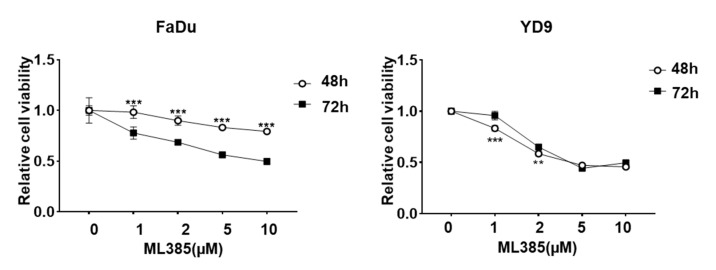
ML385 inhibits the viability of HNSCC cells. ML385 showed cytotoxic effect on HNSCC cells. Cell viability was measured using the EZ-Cytox^®^ Cell Viability assay in FaDu and YD9 cells treated with various concentrations of ML385 for 48 h (white circle) or 72 h (black square). ** *p* < 0.01, and *** *p* < 0.001.

ML385 regulates NRF2 expression in lung cancer. We investigated the effects of ML385 on the viability of NRF2-expressing HNSCC cell lines. In FaDu and YD9 cells, treatment with ML385 resulted in a dose- and time-dependent decrease in cell viability (Figure 2), whereas no significant changes were observed in the SCC9 (Supplementary Figure S1). These findings suggest that ML385 has a stronger ability to inhibit the growth and proliferation of cancer cells than Cisplatin and could be a potential new therapeutic agent. 

### 2.3. ML385 Reduces Oncogenic Properties in HNSCC

**Figure 3:**
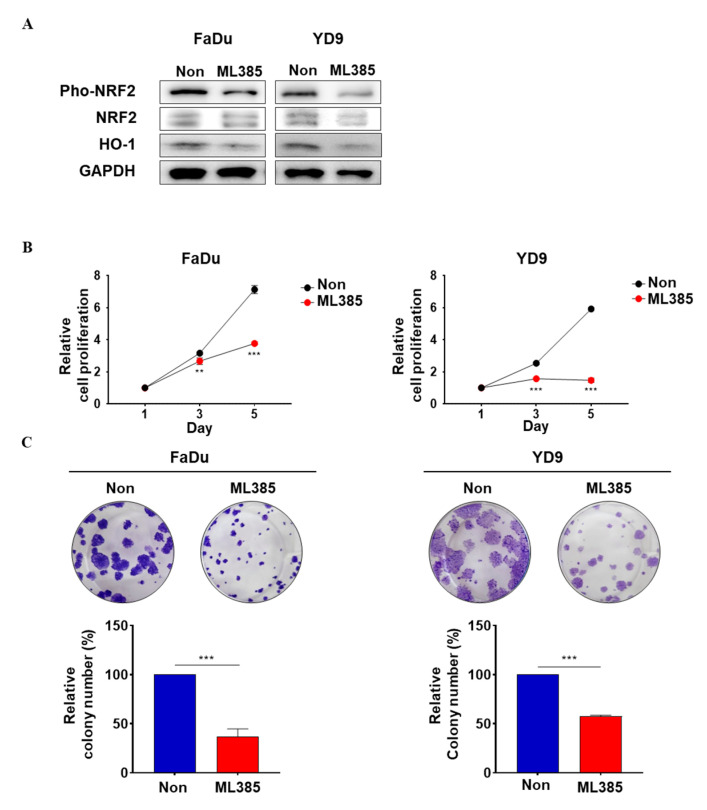

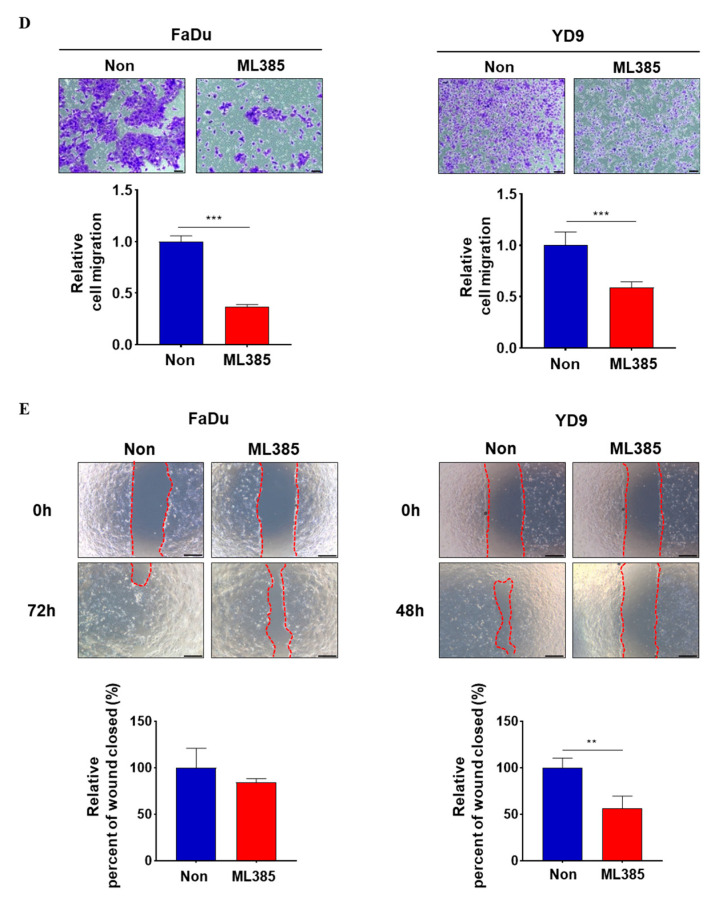
ML385 inhibits oncogenic properties. (**A**) Protein levels of ML385-treated FaDu and YD9 cells. (**B**) Cell viability was measured using the EZ-Cytox^®^ Cell Viability assay. ML385 treated cells were cultured with 5 µM ML385. (**C**) Representative image of the clonogenic assay for FaDu and YD9 cells and relative quantification of colony number. (**D**) Representative image of migration of FaDu and YD9 cells and relative quantification of the number of migrated cells. (**E**) Representative image of wound healing assay of FaDu and YD9 cells and relative quantification of the number of wound closed cells. ** *p* < 0.01, and *** *p* < 0.001.

Based on our findings, we confirmed that ML385 significantly reduced the viability of HNSCC cells. The protein levels were evaluated in ML385-treated FaDu and YD9 cells. ML385 decreased NRF2 phosphorylation and HO1 levels. This suggests that ML385 regulates NRF2 signalling (Figure 3A). We investigated whether ML385, which inhibits NRF2, could regulate the carcinogenic properties of head and neck cancer cells. Treatment of FaDu and YD9 cells with ML385 resulted in a significant decrease in cell viability, clonogenicity, migration, and wound-healing ability (Figure 3B–E). Similar results were observed in FaDu and YD9 cells transfected with siNRF2 (Supplementary Figure S2A–C). These results demonstrate that ML385 reduces the oncogenic characteristics of HNSCC cells, indicating that ML385 can inhibit the cancer-promoting properties of these cells.

### 2.4. ML385 Induces G1/S Phase Arrest in HNSCC

**Figure 4:**
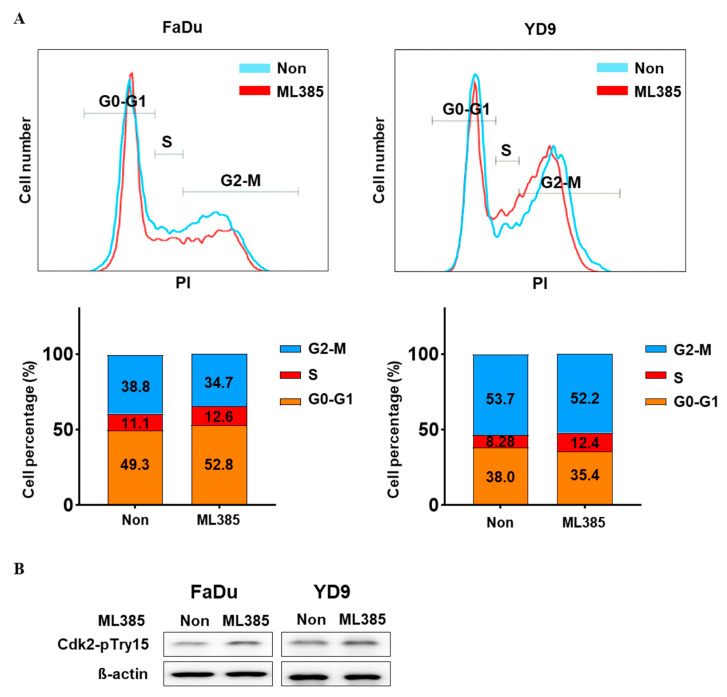
ML385 induces G1/S arrest in HNSCC cells. Flow cytometric analysis and Western blotting of ML385 treated FaDu and YD9 cells. (**A**) Flow cytometric analysis of FaDu and YD9 cells 24 h after ML385 treatment. The graph below shows the quantification of the cell cycle. (**B**) The effects of ML385 on the expression of cell-cycle-related proteins were analysed using Western blotting.

**Figure 5:**
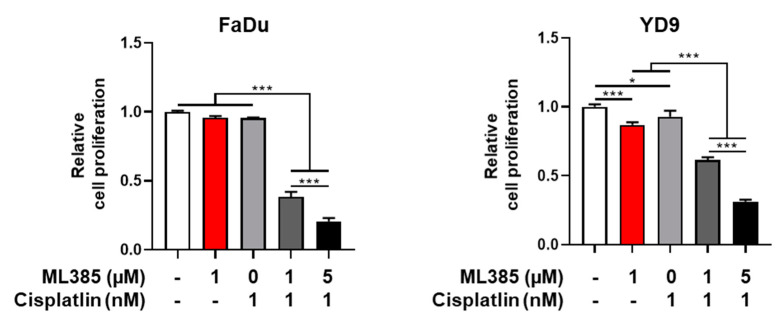
Co-treatment with ML385-Cisplatin induces apoptotic death in HNSCC cells. FaDu and YD9 cells were treated with ML385, Cisplatin, or their combination for 48 h or 72 h. Cell viability was measured using the EZ-Cytox^®^ Cell Viability assay. * *p* < 0.05 and *** *p* < 0.001.

Although ML385 treatment reduced the oncogenic properties of FaDu and YD9 cells, it did not significantly increase apoptosis (Supplementary Figure S3). To investigate the mechanism underlying the inhibitory effects of ML385 on cancer growth, cell cycle analysis was performed using FACs and Western blotting. The results revealed that both FaDu and YD9 cells were arrested in the G1/S phase (Figure 4A,B). These findings provide evidence of the ability of ML385 to disrupt normal cell cycle progression, ultimately leading to the suppression of cancer growth. We treated FaDu and YD9 cell lines with different concentrations of Cisplatin to determine their respective IC50 values, which were found to be 24.99 µM for FaDu and 8.68 µM for YD9 (Supplementary Figure S4). We then investigated the potential of combining ML385 with Cisplatin by treating the cells with 1 µM Cisplatin, which is significantly lower than the IC50 values, in combination with varying concentrations of ML385 (Figure 5). The results revealed that the survival rate of cells treated with ML385 varied depending on the dosage. This suggests that the efficacy of ML385 may be influenced by the dose used. Furthermore, when ML385 and Cisplatin were combined, a synergistic inhibitory effect on cancer cell growth was observed. The combination of ML385 and Cisplatin resulted in a significantly greater reduction in cell viability than single-agent treatment with Cisplatin. These findings suggest that the combination of ML385 and Cisplatin holds promise as a potential therapeutic approach for enhancing anticancer effects. Further studies are warranted to explore the underlying mechanisms and optimise dosing regimens for this combination therapy.

## 3. Discussion

HNSCC is a globally prevalent cancer that is associated with significant mortality and morbidity. The treatment options for HNSCC include surgery, chemotherapy, radiation therapy, targeted therapy, and immunotherapy. Owing to the unfavourable prognosis of head and neck cancer, extensive research is underway to enhance treatment outcomes using novel therapeutic agents. 

In this study, we investigated the potential of ML385, an NRF2 inhibitor, as a novel treatment for HNSCC. NRF2 is overexpressed in various malignant tumours, including head and neck cancer [19,20,21,22,23], and is associated with tumour aggressiveness, progression, and drug resistance [22]. Consequently, NRF2 has emerged as an important therapeutic target in cancer treatment. 

NRF2, also known as NFE2L2, is a key transcription factor that controls the cellular defence responses to various forms of cellular stress, including oxidative stress. Transcription factor NrF2 is a member of the cap-and-collar (CNC) or CNC-bZIP protein families [24]. KEAP1 or NRF2 inhibitors are oxidative stress-regulated substrate adaptor proteins for the Cullin-3/Rbx-1 ubiquitin ligase complex [25]. The stability of NRF2 is regulated by KEAP1, which promotes its degradation via the ubiquitin–proteasome pathway in the absence of oxidative stress [26,27,28]. The N-terminal hydrophobic region of NRF2 is followed by the INrf2 (KEAP1)-binding domain and transcriptional activation domain. NRF2-KEAP1 acts as a sensor of oxidative stress induced by chemicals and radiation. NRF2 controls numerous defensive and protective proteins in the body [29]. Under normal physiological conditions, NRF2 is detected in the cytoplasm of cells complex with KEAP1. NRF2 also affects mitochondrial functions such as apoptosis and mitochondrial biogenesis [30]. In addition, the primary role of NRF2 is to maintain the balance of mitochondrial ROS by encouraging the development of detoxification and antioxidative systems [31]. Therefore, further research is needed to investigate the potential role of the NRF2/KEAP1 pathway in interactions with anticancer drugs. 

While various agents, such as apigenin [32] and curcumin, have been reported as NRF2 inducers, there are few known NRF2 inhibitors. As the overexpression of NRF2 affects the growth of cancer cells, inhibitors can be used as new anticancer agents. ML385 is a specific NRF2 inhibitor. As a probe molecule, ML385 binds to NRF2 and inhibits downstream target gene expression. Specifically, ML385 binds to the CNC-bZIP domain of NRF2, preventing the V-Maf avian musculoaponeurotic fibrosarcoma oncogene homologue G (MAFG)–NRF2 protein complex from binding to regulatory DNA-binding sequences [33]. The small molecule ML385 was found to increase the cytotoxicity of chemotherapeutic agents in NSCLC cells, including an Nrf2-mutant LSCC cell line. It was discovered through the high-throughput screening of 400,000 small molecules by Singh. This compound has high specificity and selectivity for NSCLC, with constitutive NRF2 activation caused by inactivation mutations in KEAP1. ML385, in conjunction with carboplatin, has been shown to have a strong anticancer effect in preclinical models of NSCLC, demonstrating the potential of NRF2 as a therapeutic target for advanced NSCLC [11]. The main drug used to treat head and neck cancers is Cisplatin, which disrupts DNA synthesis and repair mechanisms, causes damage and induces cell death [13]. Recently, NRF2 inhibitors that reduce Cisplatin resistance in head and neck cancer [34] have been reported, and it is known that a decrease in the NRF2 downstream target gene HO1 contributes to the reduction of Cisplatin resistance [35].

This study confirms the potential of the NRF2 inhibitor ML385 as a therapeutic agent for head and neck cancers. The effects of the drug were observed in head and neck cancer cells expressing NRF2. We used three cell lines Fadu, YD9, and SCC9 and observed the effects of ML385 only on FaDu and YD9 cells. This difference is believed to be due to the phosphorylation of NRF2 and the differential expression of KEAP1, suggesting that KEAP1 could also be a novel therapeutic target (Supplementary Figure S5). ML385 exhibited higher responsiveness to the drug than Cisplatin and showed greater drug responsiveness in combined therapy compared to Cisplatin alone. Treatment with ML385 resulted in a decrease in NRF2 expression, which in turn reduced the expression of the downstream target gene HO1, leading to G1/S phase arrest and a decrease in the survival rate of head and neck cancer cells.

Therefore, inhibiting NRF2 rather than targeting specific downstream metabolic pathways may be more effective in the treatment of head and neck cancer. Although there are currently no FDA-approved drugs specifically targeting NRF2 in cancer, efforts to develop novel inhibitors, such as ML385, are expanding, and further research in this field is believed to have high potential for clinical applications.

This result indicated that the NRF2 inhibitor ML385 inhibited the growth of head and neck cancer cells through G1/S phase arrest. In particular, when the cells were treated with ML385 and Cisplatin in a test tube, ML385 showed better therapeutic effects than Cisplatin. This could promote the candidature of ML385 as a candidate for a new treatment. Furthermore, NRF2 presents the potential of a novel biomarker that could be targeted in head and neck cancer treatment. 

## 4. Materials and Methods

### 4.1. Gene Expression Profiling Interactive Analysis (GEPIA)

The GEPIA2 database (GEPIA 2 (cancer-pku.cn) accessed on 1 February 2024) is a web-based data-mining platform that uses large RNA sequencing data from TCGA and GTEx. GEPIA2 was used to analyse the transcript levels and survival rates of NRF2 and EGFR in human head and neck cancer. 

### 4.2. Cell Culture

FaDu cells were purchased from the ATCC (Manassas, VA, USA). The FaDu cell line was maintained in MEM (MEM, Welgene, Gyeongsan-si, Korea), supplemented with 10% fetal bovine serum (FBS, Thermo Fisher Scientific, Waltham, MA, USA), 1% penicillin-streptomycin (Thermo Fisher Scientific), and 1% sodium persulfate (Thermo Fisher Scientific) at 37 °C in a humidified atmosphere containing 5% CO_2_. The YD9 cell line was purchased from KCLB (Seoul, Korea). The YD9 cell line was maintained in Roswell Park Memorial Institute 1640 (RPMI 1640, Thermo Fisher Scientific), supplemented with 10% FBS (Thermo Fisher Scientific), 1% penicillin-streptomycin (Thermo Fisher Scientific), and 1% sodium persulfate (Thermo Fisher Scientific) at 37 °C in a humidified atmosphere containing 5% CO_2_.

### 4.3. Analysis of Cell Viability, Proliferation Rate, Migration, Colonogenicity, and Wound Healing

Cell Viability and proliferation were measured using an EZ-Cytox Cell Viability Assay Kit (Dogenbio, Seoul, Korea). FaDu and YD9 cell lines were seeded at a density of 3 × 10^3^ cells/well in 96-well plates. After overnight incubation, ML385 (#21114; Cayman, Ann Arbor, MI, USA) was added to each well and incubated for 48 h with FaDu and 72 h with YD9. Then, 10 μL EZ-Cytox was added into the well and measured at 450 nm using a microplate reader (Bio–Tek, Winooski, VT, USA). For cell migration assay, the cell lines were seeded in the upper compartment of a transwell chamber (Corning Inc., New York, NY, USA) in 200 μL of serum-free medium, and 700 μL of complement culture medium was added to the lower compartment. After 48 h, the cells remaining on the upper membrane were removed using cotton wool. The cell lines were fixed in methanol and then stained with 0.1% crystal violet (Sigma-Aldrich, St. Louis, MO, USA). For the clonogenicity assay, cell lines were seeded at a density of 300 cells/well in 6-well plates. After 14 days, cells were fixed with methanol and then stained with 0.1% crystal violet (0.1% *w*/*v*, Sigma-Aldrich). Visible colonies were counted under a microscope. For the wound-healing assay, the cell lines were seeded at 3 × 10^5^ cells per well in 24-well plates. A wound was created by scratching the confluent cell monolayers with a pipette tip. The cells were allowed to migrate for 72 h. The cell lines were fixed in methanol and then stained with 0.1% crystal violet (Sigma-Aldrich). Images of cell migration were captured using an Olympus CKX53 inverted microscope (Olympus Corporation, Tokyo, Japan).

### 4.4. Small Interfering RNA (siRNA) Transfection

FaDu or YD9 cells were transfected with siRNA for NRF2 and negative control (NC) (Bioneer, Daejeon, Korea) using the RNAiMAX transfection reagent (Thermo Fisher Scientific) according to the manufacturer’s instructions. The siRNA sequences used are listed in Table 1. 

### 4.5. Total RNA Extraction and Quantitative Reverse Transcription-PCR (qRT-PCR)

Total RNA was extracted using Nucleozol (MACHEREY-NAGEL GmbH & Co., KG, Düren, Germany) according to the manufacturer’s instructions. cDNA was synthesised with PrimeScript™ RT reagent Kit (Takara, Kusatsu, Shiga, Japan), according to the manufacturer‘s instructions. Quantitative real-time PCR was performed by a Step One Plus real-time PCR system (Applied Biosystems, Waltham, MA, USA) using Fast SYBR Green Master Mix (Applied Biosystems). *GAPDH* and *β-ACTIN* mRNA expression levels were used to normalise mRNA expression levels. Primer sequences used in this study are listed in Table 2.

### 4.6. Western Blotting

FaDu, YD9, and SCC9 cells were lysed in RIPA buffer (Thermo Fisher Scientific) containing ethylenediaminetetraacetic acid (EDTA) solution (Thermo Fisher Scientific) and phosphatase and protease inhibitors (Thermo Fisher Scientific). The lysates were incubated on ice for 30 min and centrifuged at 13,000 rpm for another 30 min. The protein concentration was measured using a BCA assay kit (Thermo Fisher Scientific). Total protein (20 μg) was separated using Mini-PROTEAN TGX Gels (Bio-Rad, Hercules, CA, USA) and electrotransferred to polyvinylidene difluoride (PVDF) membranes (Bio-Rad) using a Trans-Blot Turbo Transfer System (Bio-Rad). The membranes were blocked in 5% bovine serum albumin (BSA) at room temperature (RT) for 1 h and then incubated with a specific primary antibody overnight at 4 °C. The membranes were washed with phosphate-buffered saline (PBS) containing 0.1% Tween-20 and then probed with HRP-conjugated secondary antibodies. Protein bands were detected using ECL Prime Western blotting Detection Reagent (GE Healthcare, Chicago, IL, USA) according to the manufacturer’s instructions. The antibodies used in the experiments are listed in Table 3.

### 4.7. Apoptosis Assay

FaDu or YD9 cells were grown in complete culture media in the presence of ML385 (5 µM) for 72 h. Cells were then washed with PBS twice, and cell pellets were resuspended in 1× binding buffer to 1 × 10^5^ cells /100 μL media. Cells were incubated with 5 μL of Annexin V-FITC and propidium iodide (BD Biosciences, Franklin Lakes, NJ, USA) solutions in the dark. After 15 min, 400 μL of 1× binding buffer was added to each tube, and samples were measured by FACS Verse (BD Biosciences).

### 4.8. Cell Cycle Assay

FaDu or YD9 cells were grown in complete culture media in the presence of ML385 (5 µM) for 24 h. Cells were washed twice with PBS, pelleted, and fixed in cold 70% ethyl alcohol for at least 30 min. After being washed twice with cold PBS, cells were incubated with 50 µg/mL RNase A for 30 min at 37 °C. The cells were then stained with 100 mg/mL propidium iodide for 30 min at RT. The samples were immediately analysed using flow cytometry. The cell cycle was quantified using an FACS system (Beckman Coulter, Brea, CA, USA), according to the manufacturer’s instructions.

### 4.9. Statistical Analysis

All data were analysed using the GraphPad Prism 5.0 software (GraphPad Software, San Diego, CA, USA). Statistical tests included analysis of variance (ANOVA), Tukey’s multiple comparison test, and Student’s *t*-test. All data are presented as the mean ± SD, where indicated. The threshold for statistical significance was set at *p* < 0.05.

## Figures and Tables

**Table 1:** List of siRNA sequences used in this study.

Gene	Sense	Anti-Sense
si*NRF2* (si*NFE2L2*)	GAGACUACCAUGGUUCCAA	UUGGAACCAUGGUAGUCUC

**Table 2:** List of PCR primer sequences used in this study.

Gene	Sense	Antisence
*NRF2*	TCAGCGACGGAAAGAGTATGA	CCACTGGTTTCTGACTGGATGT
*KEAP1*	CTGGAGGATCATACCAAGCAGG	GGATACCCTCAATGGACACCAC
*HO-1*	AAGACTGCGTTCCTGCTCAAC	AAAGCCCTACAGCAACTGTCG
*GAPDH*	CTCTGCTCCTCCTGTTCGAC	TTAAAAGCAGCCCTGGTGAC
*β-ACTIN*	TCCTCTCCCAAGTCCACACAGG	GGGCACGAAGGCTCATCATTC

**Table 3:** List of antibodies used in this study.

Antibody	Company	Catalogue No.
Anti-Phosphor-NRF2	Abcam	ab76026
Anti-NRF2	Abcam	Ab62352
Anti-Cleaved CASPASE-3	Cell signalling	9661S
Anti-PARP	Cell signalling	9542S
Anti-KEAP1	Santacruz	SC365626
Anti-HO-1	Cell signalling	43966S
Cell cycle (pCdk/Phh3/actin)	Abcam	ab136810
*β-ACTIN*	Cell signalling	4967
*GAPDH*	Cell signalling	5174

## Data Availability

Data are contained within the article or Supplementary Material.

## Supplementary Materials

The following supporting information can be downloaded at: https://www.mdpi.com/article/10.3390/ijms25137011/s1.

## References

[1] Jemal A., Siegel R., Ward E., Hao Y., Xu J., Thun M.J. (2009). Cancer statistics, 2009. CA Cancer J. Clin..

[2] Warnakulasuriya S. (2009). Global epidemiology of oral and oropharyngeal cancer. Oral Oncol..

[3] Parkin D.M., Bray F., Ferlay J., Pisani P. (2005). Global cancer statistics, 2002. CA Cancer J. Clin..

[4] Ajila V., Shetty H., Babu S., Shetty V., Hegde S. (2015). Human Papilloma Virus Associated Squamous Cell Carcinoma of the Head and Neck. J. Sex. Transm. Dis..

[5] Cognetti D.M., Weber R.S., Lai S.Y. (2008). Head and neck cancer: An evolving treatment paradigm. Cancer.

[6] Wakabayashi N., Itoh K., Wakabayashi J., Motohashi H., Noda S., Takahashi S., Imakado S., Kotsuji T., Otsuka F., Roop D.R. (2003). Keap1-null mutation leads to postnatal lethality due to constitutive Nrf2 activation. Nat. Genet..

[7] Loboda A., Damulewicz M., Pyza E., Jozkowicz A., Dulak J. (2016). Role of Nrf2/HO-1 system in development, oxidative stress response and diseases: An evolutionarily conserved mechanism. Cell Mol. Life Sci..

[8] Zhang Q., Liu J., Duan H., Li R., Peng W., Wu C. (2021). Activation of Nrf2/HO-1 signaling: An important molecular mechanism of herbal medicine in the treatment of atherosclerosis via the protection of vascular endothelial cells from oxidative stress. J. Adv. Res..

[9] Hartikainen J.M., Tengstrom M., Kosma V.M., Kinnula V.L., Mannermaa A., Soini Y. (2012). Genetic polymorphisms and protein expression of NRF2 and Sulfiredoxin predict survival outcomes in breast cancer. Cancer Res..

[10] Shanmugam M.K., Nguyen A.H., Kumar A.P., Tan B.K., Sethi G. (2012). Targeted inhibition of tumor proliferation, survival, and metastasis by pentacyclic triterpenoids: Potential role in prevention and therapy of cancer. Cancer Lett..

[11] Siegel R.L., Miller K.D., Wagle N.S., Jemal A. (2023). Cancer statistics, 2023. CA Cancer J. Clin..

[12] Panieri E., Saso L. (2019). Potential Applications of NRF2 Inhibitors in Cancer Therapy. Oxid. Med. Cell Longev..

[13] Singh A., Venkannagari S., Oh K.H., Zhang Y.Q., Rohde J.M., Liu L., Nimmagadda S., Sudini K., Brimacombe K.R., Gajghate S. (2016). Small Molecule Inhibitor of NRF2 Selectively Intervenes Therapeutic Resistance in KEAP1-Deficient NSCLC Tumors. ACS Chem. Biol..

[14] Huang C.F., Zhang L., Ma S.R., Zhao Z.L., Wang W.M., He K.F., Zhao Y.F., Zhang W.F., Liu B., Sun Z.J. (2013). Clinical significance of Keap1 and Nrf2 in oral squamous cell carcinoma. PLoS ONE.

[15] Dasari S., Tchounwou P.B. (2014). Cisplatin in cancer therapy: Molecular mechanisms of action. Eur. J. Pharmacol..

[16] Kool R., Dragomir A., Kulkarni G.S., Marcq G., Breau R.H., Kim M., Busca I., Abdi H., Dawidek M., Uy M. (2024). Benefit of Neoadjuvant Cisplatin-Based Chemotherapy for Invasive Bladder Cancer Patients Treated with Radiation-Based Therapy in a Real-World Setting: An Inverse Probability Treatment Weighted Analysis. Eur. Urol. Oncol..

[17] Maeda O., Furune S., Kanda M., Miyata K., Shimizu D., Sugita S., Nishida K., Ando M., Kodera Y., Ando Y. (2024). Docetaxel, cisplatin, and fluorouracil with pegfilgrastim on day 3 as neoadjuvant chemotherapy for esophageal cancer. Cancer Med..

[18] Tchounwou P.B., Dasari S., Noubissi F.K., Ray P., Kumar S. (2021). Advances in Our Understanding of the Molecular Mechanisms of Action of Cisplatin in Cancer Therapy. J. Exp. Pharmacol..

[19] Hotter G., Rosello-Catafau J., Closa D., Bioque G., Gelpi E., Javerbaum A., Gonzalez E., Gimeno M.A. (1993). Liquid chromatography and radioimmunoassay method for the determination of prostaglandins E1 and E2 in rat embryo incubates. J. Chromatogr..

[20] Long J., Wang W., Chu J., Li Y., Wang M., Su J., Yang Y., Wang G., Li Q., Cheng H. (2024). Overexpression of Nrf2 reverses ferroptosis induced by Arenobufagin in gastric cancer. Toxicol. Appl. Pharmacol..

[21] Rahman M.M., Hossain M.M., Islam S., Ahmed R., Majumder M., Dey S., Kawser M., Sarkar B., Himu M.E.R., Chowdhury A.A. (2024). CTC together with Shh and Nrf2 are prospective diagnostic markers for HNSCC. BMC Mol. Cell Biol..

[22] Roh J.L., Kim E.H., Jang H., Shin D. (2017). Nrf2 inhibition reverses the resistance of cisplatin-resistant head and neck cancer cells to artesunate-induced ferroptosis. Redox Biol..

[23] Tuerhong A., Xu J., Wang W., Shi S., Meng Q., Hua J., Liu J., Zhang B., Yu X., Liang C. (2024). CPT1B maintains redox homeostasis and inhibits ferroptosis to induce gemcitabine resistance via the KEAP1/NRF2 axis in pancreatic cancer. Surgery.

[24] Moon E.J., Giaccia A. (2015). Dual roles of NRF2 in tumor prevention and progression: Possible implications in cancer treatment. Free Radic. Biol. Med..

[25] Namani A., Li Y., Wang X.J., Tang X. (2014). Modulation of NRF2 signaling pathway by nuclear receptors: Implications for cancer. Biochim. Biophys. Acta.

[26] Kansanen E., Kuosmanen S.M., Leinonen H., Levonen A.L. (2013). The Keap1-Nrf2 pathway: Mechanisms of activation and dysregulation in cancer. Redox Biol..

[27] Sova M., Saso L. (2018). Design and development of Nrf2 modulators for cancer chemoprevention and therapy: A review. Drug Des. Devel Ther..

[28] Taguchi K., Motohashi H., Yamamoto M. (2011). Molecular mechanisms of the Keap1-Nrf2 pathway in stress response and cancer evolution. Genes. Cells.

[29] Adinolfi S., Patinen T., Jawahar Deen A., Pitkanen S., Harkonen J., Kansanen E., Kublbeck J., Levonen A.L. (2023). The KEAP1-NRF2 pathway: Targets for therapy and role in cancer. Redox Biol..

[30] Dinkova-Kostova A.T., Abramov A.Y. (2015). The emerging role of Nrf2 in mitochondrial function. Free Radic. Biol. Med..

[31] Wang H., Liu C., Zhao Y., Zhang W., Xu K., Li D., Zhou Y., Li H., Xiao G., Lu B. (2020). Inhibition of LONP1 protects against erastin-induced ferroptosis in Pancreatic ductal adenocarcinoma PANC1 cells. Biochem. Biophys. Res. Commun..

[32] Paredes-Gonzalez X., Fuentes F., Su Z.Y., Kong A.N. (2014). Apigenin reactivates Nrf2 anti-oxidative stress signaling in mouse skin epidermal JB6 P + cells through epigenetics modifications. AAPS J..

[33] Xian P., Hei Y., Wang R., Wang T., Yang J., Li J., Di Z., Liu Z., Baskys A., Liu W. (2019). Mesenchymal stem cell-derived exosomes as a nanotherapeutic agent for amelioration of inflammation-induced astrocyte alterations in mice. Theranostics.

[34] Xu T., Yang Y., Chen Z., Wang J., Wang X., Zheng Y., Wang C., Wang Y., Zhu Z., Ding X. (2023). TNFAIP2 confers cisplatin resistance in head and neck squamous cell carcinoma via KEAP1/NRF2 signaling. J. Exp. Clin. Cancer Res..

[35] Lv X., Song D.M., Niu Y.H., Wang B.S. (2016). Inhibition of heme oxygenase-1 enhances the chemosensitivity of laryngeal squamous cell cancer Hep-2 cells to cisplatin. Apoptosis.

