# Supplementary material for: The Effects of ML385 on Head and Neck Squamous Cell Carcinoma: Implications for NRF2 Inhibition as a Therapeutic Strategy
Source: Int J Mol Sci. 2024 Jun 27;25(13):7011. doi: 10.3390/ijms25137011 (PMC11241175; doi:10.3390/ijms25137011)
Supplement: Supplementary file 1 [file ijms-25-07011-s001.zip › ijms-3057164-supplementary.pdf]

Supplementary Materials and Methods

The Effects of ML385 on Head and Neck Squamous Cell Carcinoma

# : Implications for NRF2 Inhibition as a Therapeutic Strategy

Authors: Eun-Jeong Jeong, Jong Joong Choi, Sun Young Lee, Yeon Soo Kim

Supplementary figure S1

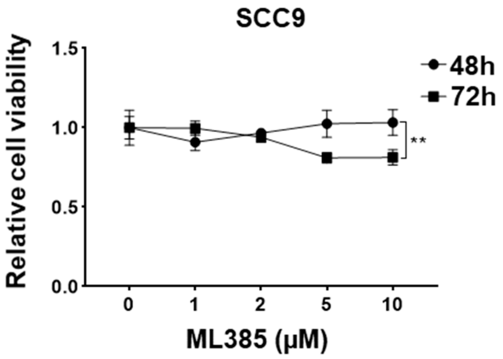

Figure S1. ML385 does not affect SCC9 cells. (A) ML385 showed cytotoxic effect on HNSCC cells. Cell viability was measured using a EZ-Cytox® Cell Viability assay in SCC9 cell treated with various concentration of ML385 for 48h (black circle) or 72h (block square). ** $p < 0.01$

Supplementary figure S2

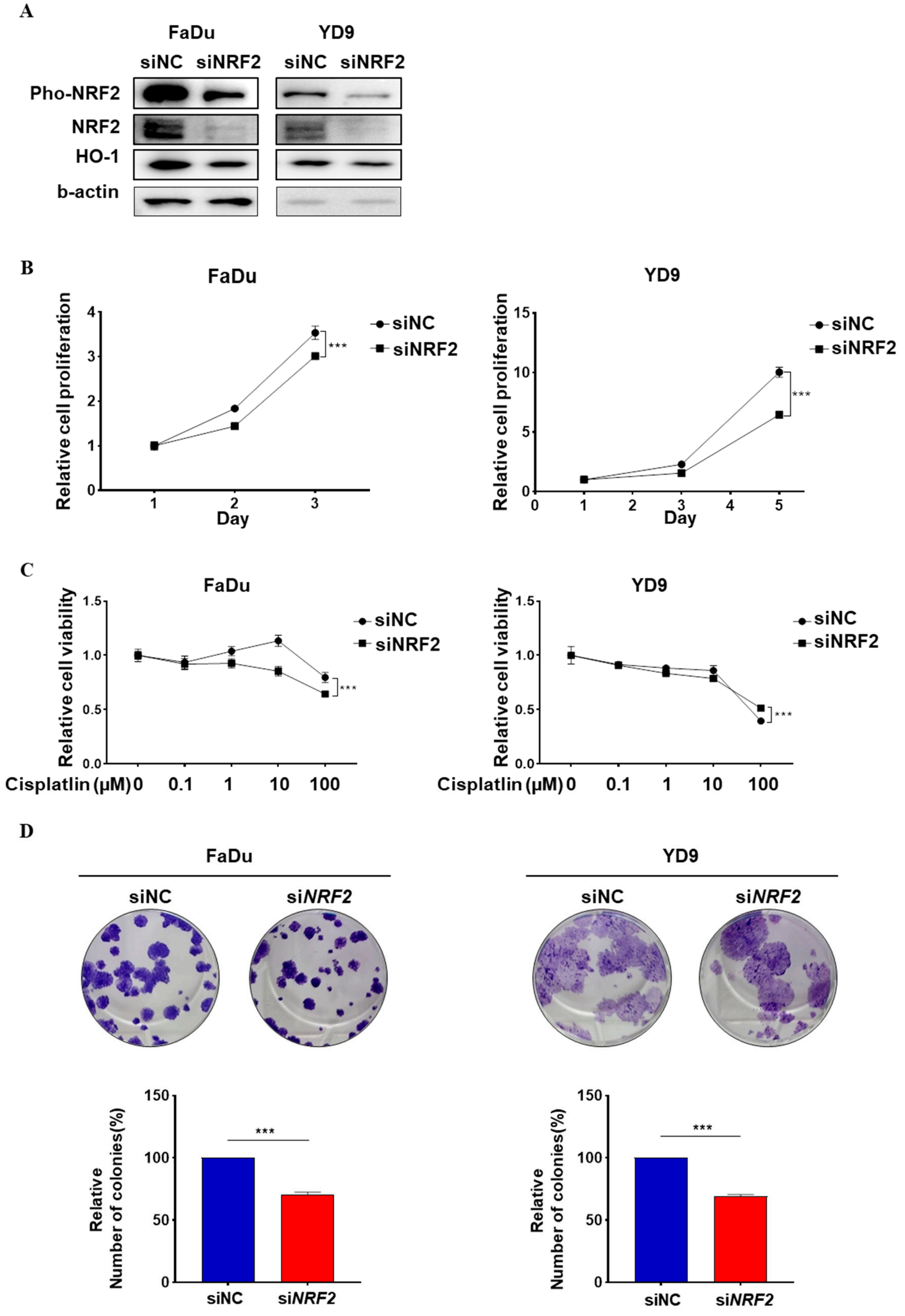

Figure S2. Confirmation of the ML385 on *NRF2*-knockdowsn FaDu and YD9 cells (A) Protein expression level of NRF2 in *NRF2*-knockdowns FaDu and YD9 cells. (B) Cell viability was measured using a EZ-Cytox® Cell Viability assay. Comparative analysis of cell proliferation between siRNA for negative control (siNC, black circle) and siRNA for NRF2 (si*NRF2*, black square) in FaDu and YD9 cells using a EZ-Cytox® Cell Viability assay. (C) Cell viability was measured using a EZ-Cytox® Cell Viability assay. *NRF2*-knockdown FaDu and YD9 cells were cultured with various concentration of cisplatin for 72h. (D) Representative image of the clonogenic assay for *NRF2*-knockdowns FaDu and YD9 cells and relative quantification of the colony number. ***$p < 0.001$

Supplementary figure S3

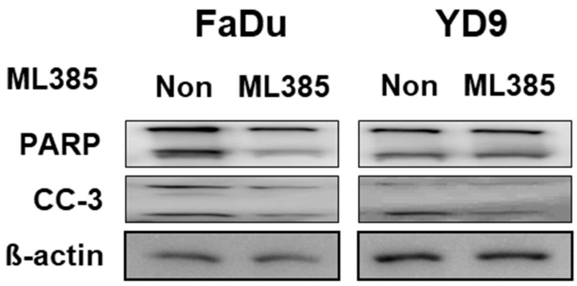

Figure S3. ML385 did not induce an increase in cleaved caspase-3 and PARP in FaDu and YD9 cells. Expression of cell death markers was determined by Western blotting.

Supplementary figure S4

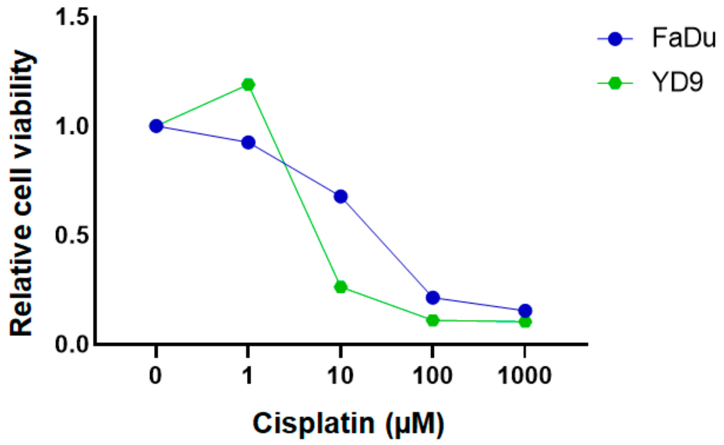

Figure S4. FaDu and YD9 cell lines were treated with different concentrations of Cisplatin, and cell viability was measured using the EZ-Cytox® Cell Viability assay. The IC50 for FaDu was 24.99 μM, and the IC50 for YD9 was 8.68 μM.

Supplementary figure S5

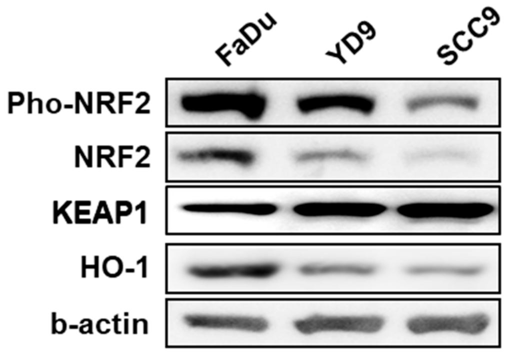

Figure S5. Confirmation of KEAP1 and NRF2 expression in HNSCC

Supplementary figure S6

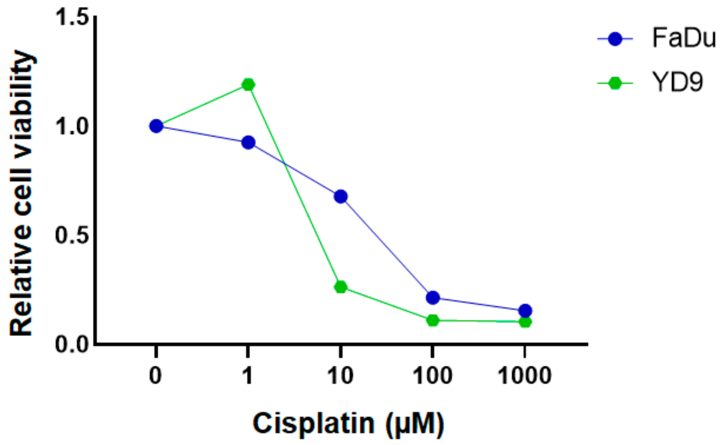

Figure S6. FaDu and YD9 cell lines were treated with different concentrations of Cisplatin, and cell viability was measured using the EZ-Cytox® Cell Viability assay. The IC50 for FaDu was 24.99 µM, and the IC50 for YD9 was 8.68 µM.
